# Differential Profile of *BRCA1* vs. *BRCA2* Mutated Families: A Characterization of the Main Differences and Similarities in Patients

**DOI:** 10.31557/APJCP.2019.20.6.1655

**Published:** 2019

**Authors:** Gabriela Carvalho Fernandes, Paula Silva Felicio, Rodrigo Augusto Depieri Michelli, Aline Silva Coelho, Cristovam Scapulatempo-Neto, Edenir Inêz Palmero

**Affiliations:** 1 *Molecular Oncology Research Center, *; 2 *Department of Oncogenetics,*; 3 *Department of Pathology, Barretos Cancer Hospital, *; 4 *Barretos School of Health Sciences, Dr. Paulo Prata – FACISB, Brazil. *

**Keywords:** Hereditary breast cancer, family history of cancer, BRCA1 vs. BRCA2 mutated patients

## Abstract

The identification of families at-risk for hereditary breast cancer (BC) is important because affected individuals present a much higher cancer risk than the general population. The aim of this study was to identify the most important factors associated with the presence of a pathogenic *BRCA1/BRCA2 *mutation. Family history (FH), histopathological and immunohistochemical characteristics were compared among BC women with pathogenic *BRCA1/BRCA2* variants; VUSs in B*RCA1/BRCA2*; *BRCA1/BRCA2 WT* and sporadic BC. The most significative differences observed concerned the molecular subtype of the tumors, age at cancer diagnosis and FH of cancer. The presence of bilateral breast cancer (BBC), number of BC cases and the presence of ovarian cancer (OC) increased (respectively) 5.797, 5.033 and 4.412 times the risk of being a *BRCA1/BRCA2* mutation carrier. Besides, women with *BRCA1* or *BRCA2* mutations presented different tumor and FH profiles. The main characteristics associated with a *BRCA1* mutation were triple negativity (OR: 17.31), BBC history (OR: 4.96) and occurrence of OC (OR: 4.32). There were no major discerning components associated with *BRCA2* mutations. Thus, we conclude that tumor pathology and FH of cancer might be considered together at the time of genetic testing mainly in countries where access to genetic testing is still restricted.

## Introduction

Cancer has been considered a public health problem for some time in developed and developing countries (UICC, 2016; WHO, 2016). Of the breast cancer (BC) cases diagnosed each year, it is estimated that 5% to 10% are inherited. The main syndrome associated with hereditary BC is known as Hereditary Breast and Ovarian Cancer Predisposition Syndrome (HBOC) (Miki et al., 1994; Wooster et al., 1994), whose main associated genes are *BRCA1* (Miki et al., 1994) and *BRCA2 *(Wooster et al., 1994). 

It is believed that the *BRCA1*/*BRCA2* genes are responsible for about 15%-25% of all cases of hereditary breast and ovarian cancer (Easton, 1999; Couch et al., 2014; Mehrgou and Akouchekian, 2016). In addition, studies have reported an increased risk of male breast cancer associated with germline mutations in *BRCA1*, although it represents a lesse frequent association than that with *BRCA2* germline mutations (Struewing et al., 1995; Milne et al., 2008). The *BRCA2* gene increases the risk of developing multiple tumors, such as: tumors of the biliary tract, bladder, esophagus, pancreas, prostate, stomach, melanoma, hematopoietic system, oral cavity and pharynx (Breast Cancer Linkage, 1999; Risch et al., 2006). 

In addition to family history, histopathological and immunohistochemical characteristics of tumors are intrinsically related to hereditary breast cancer. Women with triple negative breast cancer (TNBC) who are diagnosed at an early age are candidates for *BRCA1* genetic testing, even if they do not have a family history of breast or ovarian cancer (Lakhani et al., 2002; Oldenburg et al., 2006; Young et al., 2009). On the other side, tumors associated with germline mutations in *BRCA2* generally have immunohistochemical characteristics similar to sporadic tumors (Foulkes et al., 2003; Palacios et al., 2005). Several studies indicate that *BRCA2* tumors are more frequently luminal B subtype (Palacios et al., 2005; Bane et al., 2007; Larsen et al., 2013). Moreover, a study by Bane and colleagues reported that *BRCA2*-associated breast tumors are characterized by an increased expression of fibroblast growth factor 1 and fibroblast growth factor receptor 2 compared to *BRCA1*-associated breast tumors (Bane et al., 2009). In contrast to breast tumors associated with *BRCA1*, lack of caveolin-1 expression in breast tumors has been reported in patients with germline mutations in *BRCA2*, suggesting that the expression of caveolin-1 occurs only in tumors with mutations in *BRCA1* (Pinilla et al., 2006). 

Based on these factors, this study aimed to compare hereditary breast tumors with and without *BRCA1* and *BRCA2* pathogenic variants, with those carrying *BRCA1* and *BRCA2* variants of unknown significance (VUS) and with patients with sporadic breast cancer regarding to the family history of cancer, as well as with histopathological and imunnohistochemical tumor characteristics. In addition, *BRCA1* mutated patients were compared with *BRCA2 *mutated ones, in order to identify which are the main similarities and differences between them and which are the most important factors for the identification of a patient with a germline and pathogenic mutation in *BRCA1* or *BRCA2*.

## Materials and Methods

*Patients*

Women with a personal history of breast cancer were included in the study. They were selected and posteriorly classified based on *BRCA1*/*BRCA2* genetic testing results as follows: Women in Group 1 had a personal and family history of breast cancer with a pathogenic germline variant in *BRCA1* and/or *BRCA2*. Group 2 consisted of women with a personal and family history of breast cancer, with the presence of an identified VUS in *BRCA1* and/or *BRCA2*. Group 3 comprised women with a personal and family history of breast cancer, without a pathogenic variant and/or variant of uncertain clinical significance in *BRCA1* and/or *BRCA2*. Group 4 comprised women with a personal history of breast cancer who were not selected according to their cancer family history and who did not undergo genetic testing for analysis of germline variants in *BRCA1* and/or *BRCA2* (“sporadic”). Women from groups 1, 2, and 3 were recruited through the Oncogenetics Department of the Barretos Cancer Hospital. 

The Research Ethics Committee of the Barretos Cancer Hospital approved this project. All participants signed an informed consent form.

*Molecular Analysis*

For the analysis of mutations in *BRCA1* and *BRCA2*, a multiplex PCR amplification of all coding exons of *BRCA1* (NCBI; NM_007294.3) and *BRCA2* (NCBI; NM_000059.3) and their respective flanking intronic regions was performed. This was followed by bi-directional Sanger sequencing (ABI 3500XL, Applied Biosystems) as described elsewhere (Fernandes et al., 2016; Palmero et al., 2016a)). In addition, large rearrangements were investigated using the multiplex ligation-dependent probe amplification (MLPA) technique.

For the classification of the germline *BRCA1* and *BRCA2* variants identified ClinVar (ClinVar) and* BRCA *share databases(UMD) were used.

*Immunohistochemistry*

Monoclonal antibodies were used against ER, PR and Ki-67, CK5/6, and CK14, and a polyclonal antibody was used against HER2. For the analysis of staining, ER, PR, CK5/6, and CK14 were considered as either negative or positive. For the cell proliferation marker Ki-67, the indexes were grouped into the categories ≤14% and >14%. For the HER2 receptor, in addition to the positive and negative categories, a third category termed inconclusive was added. In these cases, fluorescent in situ hybridization (FISH) analysis using SPEC Her-2/CEN17 Dual Color Probe were performed.

*Clinical and family history data*

The clinical data of the patients included in the study were obtained from the patient’s general medical records. Family history data were obtained from the medical records of the Oncogenetics Department of the Barretos Cancer Hospital.

*Statistical Analysis*

The program Statistical Package for Social Sciences v.21.0 for Windows (Chicago, IL) was used for the statistical analysis. Categorical variables were described using absolute frequencies and relative percentage frequencies. Correlations were performed using Chi-square and Fisher’s exact tests. Bonferroni correction for multivariate analysis was used to estimate the predictive effects of the significantly associated factors for predicting the probability of *BRCA1*/*BRCA2* mutations. The level of significance adopted in all tests was 5%.

## Results

*General Characteristics*

Patients included in the study were women with a personal history of breast cancer. Group 1 had 51 patients, Group 2 comprised 53 women, Group 3 comprised 100 women, and Group 4 had 83 women. 

The average age at diagnosis was 41.88 years (SD = 10.5 years) in Group 1, 34.91 years (SD = 10.0 years) in Group 2, 38.37 years (SD = 10.8 years) in Group 3, and 51.65 years (SD = 9.9 years) in Group 4. Detailed age distribution can be found in Supplementary Table 1. With regard to hormonal risk factors for breast cancer, we observed that 23.6% of the women were menopausal at diagnosis, and 79.4% had a previous pregnancy. The pathological data of the samples are summarized in Supplementary Table 2. Patients in Group 1 had a higher proportion of histological grade III and T4 tumors when compared to the patients from Groups 2–4. As was expected, most of the women in Group 1 had TNBC. In addition, when Group 1 was stratified according to mutated gene, we observed that, among the triple negative tumors, 91.6% had a pathogenic mutation in *BRCA1*, and only 8.4% in *BRCA2*.

When histological analysis was performed dividing Group 1 by mutated gene, we observed a small difference in histological grade, namely, 56.5% of women with a germline mutation in *BRCA1* had histological grade III tumors, compared to 25.0% of women with a germline mutation in *BRCA2* (p = 0.03). The other variables (size tumor, lymph node, metastasis) were also analyzed according to the mutated gene, but no statistically significant difference was found.

The majority of the breast tumors were ductal infiltrating carcinomas (236 cases, 91.8%), followed by lobular carcinomas (10 cases, 3.9%), and medullary carcinomas (4 cases, 1.6%). Medullary carcinomas were further investigated and it was found that two of them belonged to Group 1, one of whom had a mutation in *BRCA1*, and the other in *BRCA2*. The other two patients with medullary carcinoma were from Groups 2 and 4 respectively. 

The results of the immunohistochemistry are reported in Supplementary Table 3. A higher positivity of CK5/6 and CK14 staining was observed in Group 1 compared to the other groups, and, once more, this increase in positivity was mainly associated with *BRCA1* pathogenic variants. 

The predominant molecular subtype in all of the groups, except for those *BRCA1* mutated inside group 1, was luminal B without HER2 expression. As expected, among the group with sporadic breast cancer, the second most common subtype was luminal A. Moreover, a substantial number (30%) of tumors from Group 1 were classified as basal-like (88.8% of them with a germline mutation in *BRCA1*) (Table 1).

Regarding the expression of the evaluated cytokeratins, the cancers associated with a germline pathogenic variant in *BRCA1* were more frequently positive for CK5/6 and CK14 than tumors associated with a germline pathogenic variant in *BRCA2* (p = 0.031 and p = 0.008, respectively).

*Family history of cancer*

The pedigree of the 204 families at-risk for hereditary breast cancer included in the study were criteriously revised (Supplementary Table 1). Through the analyzed family history, it was observed that the majority of women in Group 1 reported more than three cases of breast cancer in the family (28 participants, 54.9%), while most of women in the other analyzed groups reported fewer than three cases of breast cancer (p < 0.001). As expected, the presence of bilateral breast cancer was reported most by patients in Group 1 with 10 cases (19.6%), whereas only 5 patients in Group 2 and 5 patients in Group 3 reported the presence of bilateral breast cancer (p < 0.001). As it would be expected, no patients in Group 4 were diagnosed with bilateral breast cancer, male cancer or family history with more than 3 breast cancer cases. 

In addition, the presence of ovarian cancer in the family history was observed more frequently in Group 1 (13 cases, 25.5%) than in groups 2 and 3, and no patients in Group 4 reported the presence of ovarian cancer in their families (p < 0.001). It should be noted that patients from Group 4 were not tested for germline mutations in *BRCA1*/*BRCA2* because of the criteria adopted by the Oncogenetics Department of Barretos Cancer Hospital (Palmero et al., 2016b). 

*Comparison of patients with BRCA1/BRCA2 mutations versus patients with WT BRCA1/BRCA2 and sporadic breast cancer cases regarding histopathological, molecular, and family history*

In order to identify which characteristics were typical and representative of the patients with *BRCA1/BRCA2* pathogenic germline variants, a multivariate analysis, in which the *BRCA1/BRCA2* mutation carriers were compared with the other three groups was performed. However, as observed previously, there were major differences between patients with *BRCA1* and *BRCA2* pathogenic variants. For this reason, the comparisons were performed separately for both groups. Detailed results can be found in Supplementary Tables 4 and 5 for *BRCA1* and *BRCA2* carriers, respectively. 

In relation to the family history, some variables that showed significance in the compared groups should be highlighted, such as the presence of bilateral breast cancer, ovarian cancer, number of generations affected by cancer, number of breast cancer cases and age at diagnosis. Regarding tumor characteristics, the essential variables that came out are ER, PR, HER2, and cytokeratin expression (Supplementary Tables 3 and 4).

**Figure 1:**
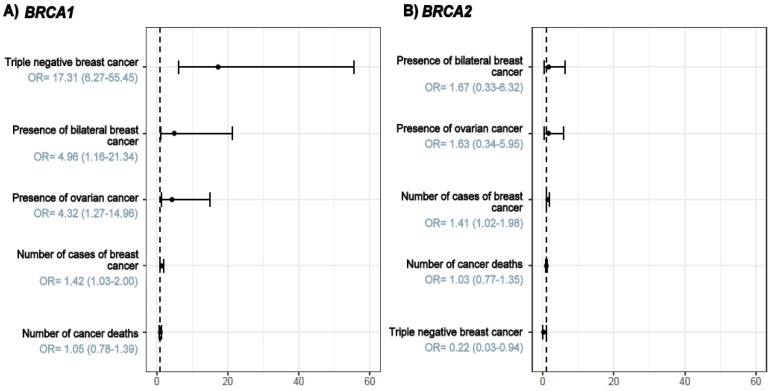
Main Factors Associated with the Presence of a *BRCA1* (in A) or *BRCA2* (in B) Pathogenic Variant

After that, a multivariate analysis – logistic regression was performed and allow the identification of the central characteristics that differed between patients with and without mutations and to see the “weight” that each of these characteristics conferred upon the likelihood of carrying a germline mutation in *BRCA1* or *BRCA2* (Figure 1). Comparing the *BRCA1* (A) and *BRCA2* (B) carriers according to personal/family history, it was possible to see that triple negativity, bilateral breast and ovarian cancer in the proband or family were the main factors associated with the presence of a *BRCA1* mutation. For *BRCA2*, there were no variables in the family history or tumor profile conferring a significative higher risk to be a carrier, highlighting how different the tumors of individuals carrying germline mutations in *BRCA1* and *BRCA2* are, in spite of their association with the same cancer predisposition syndrome, HBOC and which are the main differences and similarities between them.

**Table 1:** Molecular Subtype of Breast Tumors by Group

Variable	Group 1 N (%)		Group 2 N (%)	Group 3 N (%)	Group 4 N (%)	p-value
	MUTATED BRCA1	MUTATED BRCA2				
Molecular subtype						**
Luminal A	1 (7.7)	0 (0.0)	1 (2.6)	8 (11.0)	19 (26.0)	
Luminal B (HER2 negative)	4 (30.8)	12 (70.6)	22 (57.9)	25 (34.2)	31 (42.5)	
Luminal B (HER2 positive)	0 (0.0)	4 (23.5)	9 (23.7)	19 (26.0)	12 (16.4)	
HER2 overexpressed	0 (0.0)	0 (0.0)	5 (13.2)	11 (15.1)	7 (9.6)	
Basal-like	8 (61.5)	1 (5.9)	1 (2.6)	10 (13.7)	4 (5.5)	

**, Given the very low sample size in some of the groups, it was not possible to determine statistical significance.

## Discussion

A family history of cancer in first-degree relatives and the presence of some specific risk factors, such as bilateral breast cancer, family history of breast and ovarian cancer, and breast cancer in a male person are important indicators of risk for hereditary breast cancer. Advances in molecular biology in recent decades have resulted in the identification of genes, such as the tumor suppressor genes *BRCA1* and *BRCA2*, that, when altered, significantly increase the risk of developing breast cancer, ovarian cancer, and other tumors. Additionally, especially in developing and undeveloped countries, access to testing is still restricted owing to its high cost and the lack of coverage by health plans; therefore, the correct identification of individuals and families that would benefit from genetic testing is extremely important (Palmero et al., 2016b). 

In this study, we included 287 women with breast cancer from Barretos Cancer Hospital. The main goal of this study was to identify the principal characteristics that differentiate a family with *BRCA1*/*BRCA2 *pathogenic mutations from those with a VUS alteration and even from those *BRCA1/BRCA2 WT* and with sporadic breast tumors. For that, clinical data, tumor profile and family history of cancer were compared among the women assigned to each of the four main groups of the study. However, we find out that the variables typical of *BRCA1* carriers could not be applied to identify *BRCA2 *carriers.

The most common histological type diagnosed among the women from all four grous was invasive ductal adenocarcinoma (84.4%). Even for women with an identified germline mutation, the presence of medullary carcinoma was low (1.6%). The CIMBA group analyzed 4,325 mutation carriers and found that medullary tumors were 9.4% and 2.2% of tumors identified among *BRCA1* and* BRCA2* mutation carriers (Mavaddat et al., 2012). No difference in histological grade was observed among the four groups of patients. However, it is interesting to point that, in Group 1, a significant number of cases were grade III (43.6%). Moreover, when stratified according to the mutated gene, we observed that the majority of those grade III cases harbored *BRCA1* mutations (56.5% vs. 25.0% *BRCA2 *mutations). Although in a minor proportion, our data are in accordance with the results published by the CIMBA group, where the authors reported that 77.0% of women with mutations in *BRCA1* had grade III breast tumors, compared to only 50.0% of women with mutations in *BRCA2* (Mavaddat et al., 2012). 

Regarding the hormone receptors (ER, PR), most tumors from *BRCA1* mutated patients in Group 1 were negative for both ER and PR, unlike the other groups and also the* BRCA2* mutated from group 1, where positivity for both receptors prevailed. Most patients, regardless of group, were HER2-negative. When stratifying the women from Group 1 according to the mutated gene, we observed that 88.9% of the women with TNBC were *BRCA1* mutation carriers, while only 11.1% had a *BRCA2* mutation. These data corroborate previous findings in the literature, which show a higher incidence of triple negativity in women with a deleterious *BRCA1* mutation (Bayraktar et al., 2011; Evans et al., 2011; Hartman et al., 2012; Meyer et al., 2012; Triantafyllidou et al., 2015; Wong-Brown et al., 2015). In addition, several studies have identified individuals with *BRCA1/BRCA2* germline mutations through the analysis of TNBC cases. The rate of *BRCA1/BRCA2* mutation detection in those studies, selected based on the tumor triple negativity, independent of family history, varies from 17.4% to 49.1% (Hartman et al., 2012; Couch et al., 2015). Besides, our data on the sporadic group (Group 4) supports the findings from the literature, with a 15% to 20% frequency of TNBC(Bauer et al., 2007; Blows et al., 2010; Lin et al., 2012). 

For cases in which the result of immunohistochemistry could be obtained, we conducted a classification of molecular subtype as described by Goldhirsch(Goldhirsch et al., 2011). The data from this analysis showed that 30% of women with a germline mutation in *BRCA1* or* BRCA2* had a basal-like molecular subtype, which is lower than what has been described in the literature (Andres et al., 2014). However, when analyzing *BRCA1* mutation vs. *BRCA2* mutation, we observed that 88.8% of women with a germline mutation in *BRCA1* displayed basal-like histology versus 11.2% of those with a germline mutation in *BRCA2*. A study conducted by Pinilla et al. also reported an increased frequency of basal-like tumors in patients with *BRCA1* mutations (55.6%) compared to *BRCA2* mutation carriers (10%) (Pinilla et al., 2006).

Similar to the results of other studies, we observed an association between germline mutations in *BRCA1/BRCA2* and a family history of cancer. When comparing the presence/absence of a germline mutation with a family history of cancer, we noted that 54.9% of women in Group 1 had more than three cases of breast cancer in their family history. In addition, the presence of bilateral breast cancer was most reported by patients with a germline mutation (19.6%) compared to women without a germline mutation. This association was observed in other studies, as described by Gershoni-Baruch et al., (1999), in which 45% of patients with a germline mutation (*BRCA1/BRCA2*) and positive family history were diagnosed with bilateral breast cancer (14/31). Regarding the difference found in the family history of women with mutations in *BRCA1 *or *BRCA2* genes, we can observe that BRCA1 mutated families showed a higher frequency of bilateral breast cancer (OR=4.96 vs. OR=1.67) and presence of ovarian cancer (OR=4.32 vs. OR=1.63). 

It is noteworthy that a family history of cancer is the main indicator for genetic testing. However, several studies have pointed to the fact that many women are candidates for genetic testing by combining tumor characteristics, such as histopathology and immunohistochemistry and factors related to the personal and family history of cancer (Mavaddat et al., 2012; Spurdle et al., 2014). Taking this into consideration, a multivariate analysis was performed to identify the main characteristics associated with the presence of *BRCA1* or *BRCA2 *mutations. This analysis allowed us to identify that BRCA1 was mainly correlated with triple negativity (OR: 17.31), presence of bilateral breast cancer history (OR: 4.96), occurrence of ovarian cancer (OR: 4.32) and presence of more than three breast cancer cases in the family (OR: 1.42). When only *BRCA2 *mutation carriers were considered, there were no major discerning characteristics. These results reinforce the necessity that family history should be considered, but not in isolation, as a factor of selection and identification of families to be referenced for genetic testing. A more detailed investigation would increase the rate of detection of* BRCA1/BRCA2* mutation carriers, allowing health care providers to direct resources and expand genetic testing access to those families that have higher risk and probability of having a pathogenic *BRCA* mutation, which is very important, particularly in those countries where genetic testing is still restricted.

Finally, it is worth noting that despite the relatively restrictive criteria applied by the Oncogenetics Department for the selection of patients who should be tested and the broad methodology for the analysis of genes involved, the high/moderate risk of cancer attributed to family (in the case of Groups 2 and 3 of this study) remains unexplained. Part of this may be due to the fact that there may be genetic alterations in other genes not yet associated with hereditary breast cancer or the presence of genetic alterations in other high/moderate risk genes for which the patients were not tested (such as *TP53* and *PALB2*). In addition, it emphasizes the importance of characterizing clinical, pathological, and molecular data, including other variables, as well as family history of cancer, as a criterion for the selection/identification of women and families at-risk for hereditary breast cancer. In conclusion, this research combines both pedigree and tumor data to identify the main variables associated with the presence of a *BRCA1* or *BRCA2* germline mutation. Prediction of *BRCA1/BRCA2* carrier status, and hence selection of women for mutation screening, may be substantially improved by combining tumor pathology with family history. These variables must be considered together at the time of genetic counseling mainly in countries where access to genetic testing is still restricted. 

## Funding

This work was supported by a grant from Conselho Nacional de Desenvolvimento Científico e Tecnológico (CNPq), Edital MCT/CNPq 14/2010, process 480760/2010-1. EIP receives a National Council of Technological and Scientific Development (CNPq) scholarship.

## Competing interests

The authors declare that they have no competing interests.
